# The Criminal Type

**Published:** 1895-10-05

**Authors:** 


### Social Problems.

### THE CRIMINAL TYPE.

The study of the criminal in regard to his mental and physical development is comparatively new, and has never for some reason taken very firm root in England. The time-honoured view of punishment as "the gratification of the passion of moral indignation," whose sole legitimate end is deterrence, has, perhaps, stood in the way of investigation on the part of the authorities, and outside inquirers have been baffled by the wholesome reluctance to allow prisoners to become objects of even scientific curiosity. For these reasons investigations made in other countries where the like impediments to this form of research do not exist, have a special value, and the results of Professor Lombroso's examination of the female prisoners in France and Russia will be read with much interest.* The data, though very exact as far as they go, do not represent a very wide field of observation, seldom exceeding 1,000, and often resting on no more than twenty specimen cases, and the conclusions cannot therefore be regarded with any certainty. But the smallest glimmer of light in so dim a region is not to be despised. Professor Lombroso's theory is briefly, that the criminal type is a recurrence to old ancestral forms of low development, "a product of pathological and atavistic anomalies;" the criminal in fact "stands midway between the lunatic and the savage." The theory is built up on the observation, not of marked peculiarities stamping the offender with a brand easy to be recognised, but on countless small deviations from the normal type, shared by the criminal population, it is true, in common with many law abiding individuals, but in a far higher percentage, and especially significant in combination. "Where a considerable number of deep-seated physical anomalies are found in combination in the same individual, we usually see that they are accompanied by nervous and mental anomalies of a more or less morbid character."

The actual physical peculiarities observed among female prisoners are not very numerous or striking. Among them may be mentioned heavy jaws and high-cheek bones. Stature, strength of arms, and length of limbs was found to be below the average, and though the facial diameter was larger, the cranial diameter was considerably less than in normal subjects. Much of the evil appears to be due to the brain. The post-mortem examination of thirty-three revealed in eleven out of the number "grave macroscopic lesion of the central system and its molucra." Passing to skull anomalies, they were found less frequent among female than among male criminals, always excepting the skulls of murderesses, which are peculiar. The skull of Charlotte Corday is cited in this connection as displaying very striking irregularities.

The following small anomalies are among those which have been observed to recur among criminal and fallen women. Facial asymmetry, or a striking want of correspondence between the two sides of the face, has been noticed in 7·7 per cent.; irregularity in the shape of the ears is twice as common among criminals, and projecting ears appear to be more especially characteristic of the swindler and the poisoner; a crooked nose may be noted among one out of every four evil-doers, while though flat nose is more distinctive of the law-abiding citizen, it is a defect shared in common with a large proportion of incendiaries. A virile physiognomy, combined often with the voice and larynx of a man, were observed also in a large number of female offenders, and certain distinctly degenerate types, such as the cast of face known as the Mongolian physiognomy, and hypertrophy of the muscles of the neck, observable in large quadrupeds, were not wanting. Cleft palate, hare lip, left-handedness, anomalous teeth, though common enough among normals, were found to be twice as frequent among criminals.

The sense of touch, taste, smell, and hearing were experimented on by consent of the prisoners, and were found to be considerably less acute than in normal subjects. In sense of smell especially the criminal class seems to be singularly deficient, and only three out of fifteen of the born criminals had a normal field of vision. All these anomalies are far less prominent and frequent in females than in males, and the true criminal type is comparatively seldom encountered in women.

Some of Professor Lombroso's observations are more in the nature of sweeping generalisations than deductions from accurately observed facts. He classifies the occasional offender as "women in whom circumstances have developed the fund of immorality which is latent in every female," and has leaped to the ungallant conclusion "that in the normal female the sense of property is not strong." This painful allegation rests on the assertion that lost property restored to the municipal authorities in Paris is almost always brought back by men. A "cultivated lady," too, whose name is discreetly shrouded under an initial, once confided to him "that it is very difficult for women to play without cheating;" and, to crown all, Parisian detectives estimate the number of well-to-do female thieves unable to resist the temptation of appropriating articles which take their fancy, at from 50 to 99 per cent. of the whole, including in the number a large proportion of ladies in society. It is, however, less easy to test a "sense of property" than a sense of smell, and the "normal female" need not lay this rather vague aspersion on her morals very much to heart.

It may be asked what good can result from all this laborious classification of minute characteristics. Much of it, doubtless, is over-elaborated and beside the mark, much is disputed ground, the records of different observers varying in many important points. But the broad facts remain that certain children are born into the world with certain well-defined traits of mind and body distinct from their fellows, and that of these children a large proportion are found in later life to have run off the track and become absorbed in the criminal class. A recent investigation in London schools has shown that the number of these children amounts to 18 per cent. Is there not some reason to believe that wise treatment and special training *from the beginning* might bring under control the passions of which the bodily anomalies present a faint and often erring index, and save many lives from mischief and ultimate despair?

* "The Female Offender." By Professor Lombroso and William Ferrero. With an introduction by W. Douglas Morrison, H.M. Prison, Wandsworth. (T. Fisher Unwin.)

